# 2-Chloro-5-(2-iodo­benzene­sulfonamido)­benzoic acid

**DOI:** 10.1107/S1600536808043869

**Published:** 2009-01-10

**Authors:** Muhammad Nadeem Arshad, M. Nawaz Tahir, Islam Ullah Khan, Waseeq Ahmad Siddiqui, Muhammad Shafiq

**Affiliations:** a Government College University, Department of Chemistry, Lahore, Pakistan; b University of Sargodha, Department of Physics, Sagrodha, Pakistan; c University of Sargodha, Department of Chemistry, Sagrodha, Pakistan

## Abstract

In the mol­ecule of the title compound, C_13_H_9_ClINO_4_S, the coordination around the S atom is distorted tetra­hedral. The aromatic rings are oriented at a dihedral angle of 74.46 (9)°. Intra­molecular C—H⋯O hydrogen bonds result in the formation of two five- and one six-membered rings, which adopt planar, envelope and twisted conformations, respectively. In the crystal structure, inter­molecular N—H⋯O and O—H⋯O hydrogen bonds link the mol­ecules to form *R*_2_^2^(8) ring motifs, which are further linked by C—H⋯O hydrogen bonds. π–π contacts between the benzene rings [centroid–centroid distances = 3.709 (3) and 3.772 (3) Å] may further stabilize the structure. The I atom is disordered over two positions, refined with occupancies of *ca* 0.75 and 0.25.

## Related literature

For related structures, see: Arshad, Tahir, Khan, Ahmad & Shafiq (2008 ▶); Arshad, Tahir, Khan, Shafiq & Siddiqui (2008 ▶); Arshad *et al.* (2009 ▶); Deng & Mani (2006 ▶). For ring motifs, see: Bernstein *et al.* (1995 ▶).
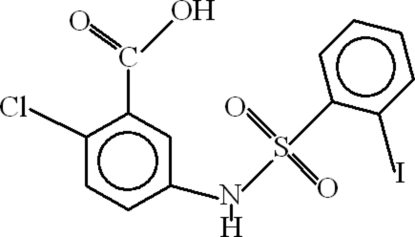


## Experimental

### 

#### Crystal data

C_13_H_9_ClINO_4_S
*M* *_r_* = 437.62
Monoclinic, 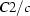

*a* = 26.6375 (9) Å
*b* = 8.5532 (2) Å
*c* = 14.2696 (5) Å
β = 111.923 (2)°
*V* = 3016.03 (17) Å^3^
*Z* = 8
Mo *K*α radiation
μ = 2.45 mm^−1^
*T* = 296 (2) K
0.25 × 0.12 × 0.08 mm

#### Data collection

Bruker Kappa APEXII CCD diffractometer
Absorption correction: multi-scan (*SADABS*; Bruker, 2005 ▶) *T*_min_ = 0.708, *T*_max_ = 0.819
16794 measured reflections
3738 independent reflections
2909 reflections with *I* > 2σ(*I*)
*R*_int_ = 0.023

#### Refinement

*R*[*F* ^2^ > 2σ(*F* ^2^)] = 0.026
*wR*(*F* ^2^) = 0.069
*S* = 1.05
3738 reflections
203 parameters
H atoms treated by a mixture of independent and constrained refinement
Δρ_max_ = 0.54 e Å^−3^
Δρ_min_ = −0.31 e Å^−3^

### 

Data collection: *APEX2* (Bruker, 2007 ▶); cell refinement: *SAINT* (Bruker, 2007 ▶); data reduction: *SAINT*; program(s) used to solve structure: *SHELXS97* (Sheldrick, 2008 ▶); program(s) used to refine structure: *SHELXL97* (Sheldrick, 2008 ▶); molecular graphics: *ORTEP-3 for Windows* (Farrugia, 1997 ▶) and *PLATON* (Spek, 2003 ▶); software used to prepare material for publication: *WinGX* (Farrugia, 1999 ▶) and *PLATON*.

## Supplementary Material

Crystal structure: contains datablocks global, I. DOI: 10.1107/S1600536808043869/hk2607sup1.cif

Structure factors: contains datablocks I. DOI: 10.1107/S1600536808043869/hk2607Isup2.hkl

Additional supplementary materials:  crystallographic information; 3D view; checkCIF report

## Figures and Tables

**Table 1:** Hydrogen-bond geometry (Å, °)

*D*—H⋯*A*	*D*—H	H⋯*A*	*D*⋯*A*	*D*—H⋯*A*
N1—H1⋯O4^i^	0.86	2.07	2.903 (3)	164
O3—H3O⋯O2^ii^	0.76 (4)	1.95 (4)	2.714 (3)	176 (5)
C4—H4⋯O1^iii^	0.93	2.48	3.293 (4)	146
C6—H6⋯O1	0.93	2.36	2.792 (3)	108
C8—H8⋯O1	0.93	2.57	3.193 (3)	125
C8—H8⋯O3	0.93	2.28	2.631 (3)	102

Symmetry codes: (i) 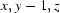; (ii) 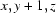; (iii) 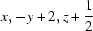.

**Fig. 1.**
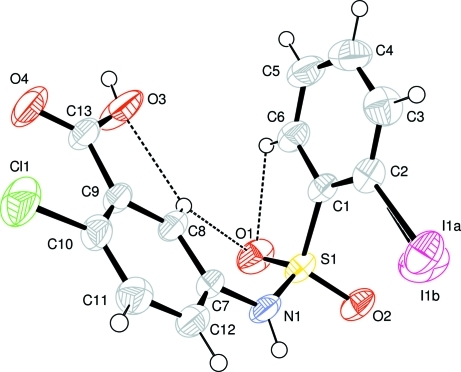
The molecular structure of the title molecule, with the atom-numbering scheme. Displacement ellipsoids are drawn at the 50% probability level. Hydrogen bonds are shown as dashed lines.

**Fig. 2.**
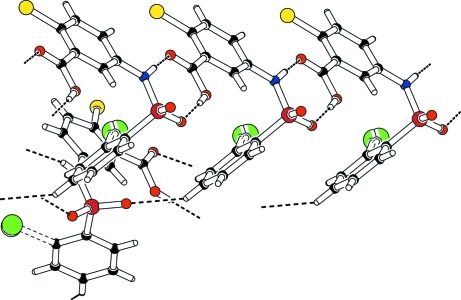
A partial packing diagram of the title compound. Hydrogen bonds are shown as dashed lines.

## supplementary crystallographic information

### Comment

In continuation to our researches with sulfonamides (Arshad, Tahir, Khan, Ahmad & Shafiq, 2008; Arshad, Tahir, Khan, Shafiq & Siddiqui, 2008; Arshad *et al.*, 2009), the title compound has been synthesized, and we report herein its crystal structure.

The structure of the title compound, (I), (Fig 1), differs from 4-[(2-iodo- phenyl)sulfonyl]aminobenzoic acid hydrate, (II) (Arshad *et al.*, 2009), due to the attachment of Cl atom and the change of the position of carboxylate group. Also in (I), there is no water molecule. The coordination around the S atom is a distorted tetrahedral. Rings A(C1-C6) and B(C7-C12) are oriented at a dihedral angle of 74.46 (9)°, which is nearly the same with the corresponding value [74.18 (17)°] in (II). The intramolecular C-H···O hydrogen bonds (Table 1) result in the formations of two five- and one six-membered rings: C (S1/O1/C1/C6/H6), D (O3/C8/C9/C13/H8) and E (S1/O1/N1/C7/C8/H8). Ring C is planar. Ring D adopts envelope conformation with O3 atom displaced by -0.260 (4) Å from the plane of the other rings atoms, while ring E has twisted conformation. The dihedral angle between rings A and C is 2.18 (3)°.

In the crystal structure, intermolecular N-H···O and O-H···O hydrogen bonds (Table 1) link the molecules to form *R*_2_^2^(8) ring motifs (Bernstein *et al.*, 1995), they are further linked by C-H···O hydrogen bonds (Table 1, Fig. 2), in which they may be effective in the stabilization of the structure. The π-π contacts between the phenyl rings and the benzene rings, Cg1—Cg1^i^ and Cg2—Cg2^ii^ [symmetry codes: (i) 1/2 - x, 3/2 - y, 1 - z; (ii) -x, 2 - y, -z, where Cg1 and Cg2 are centroids of the rings A (C1-C6) and B(C7-C12), respectively] may further stabilize the structure, with centroid-centroid distances of 3.709 (3) Å and 3.772 (3) Å.

### Experimental

The title compound was synthesized according to a literature method (Deng & Mani, 2006). 5-Amino-2-chlorobenzoic acid (0.28 g, 1.66 mmol) was suspended in distilled water (10 ml) in a round bottom flask. The pH of the solution was adjusted to 8-9 using Na_2_CO_3_ (1 M). Then, 2-iodobenzene sulfonyl chloride (0.5 g, 1.66 mmol) was added, and stirred at room temperature. The reaction pH was maintained at 8-9. Completion of reaction was indicated by the dissolvation of the suspended 2-iodobenzene sulfonyl chloride. Then, pH was adjusted to 2-3 using HCl (2 N). The precipitate formed was filtered, washed with distilled water, and then recrystalyzed in methanol.

### Refinement

The iodine atom was disordered over two positions. During the refinement process the disordered atoms I1A and I1B were refined with occupancies of 0.75 and 0.25, respectively. H3O (for OH) atom was located in difference syntheses and refined [O-H = 0.76 (4) Å, U_iso_(H) = 1.2U_eq_(O)]. The remaining H atoms were positioned geometrically, with N-H = 0.86 Å (for NH) and C-H = 0.93 Å for aromatic H, respectively, and constrained to ride on their parent atoms with U_iso_(H) = 1.2U_eq_(C,N).

### Crystal data

**Table:**

C_13_H_9_ClINO_4_S	*F*(000) = 1696
*M**_r_* = 437.62	*D*_x_ = 1.928 Mg m^−^^3^
Monoclinic, *C*2/*c*	Mo *K*α radiation, λ = 0.71073 Å
Hall symbol: -C 2yc	Cell parameters from 3738 reflections
*a* = 26.6375 (9) Å	θ = 2.5–28.3°
*b* = 8.5532 (2) Å	µ = 2.45 mm^−^^1^
*c* = 14.2696 (5) Å	*T* = 296 K
β = 111.923 (2)°	Needle, light brown
*V* = 3016.03 (17) Å^3^	0.25 × 0.12 × 0.08 mm
*Z* = 8	

### Data collection

**Table:**

Bruker Kappa APEXII CCD diffractometer	3738 independent reflections
Radiation source: fine-focus sealed tube	2909 reflections with *I* > 2σ(*I*)
graphite	*R*_int_ = 0.023
Detector resolution: 7.40 pixels mm^-1^	θ_max_ = 28.3°, θ_min_ = 2.5°
ω scans	*h* = −34→35
Absorption correction: multi-scan (*SADABS*; Bruker, 2005)	*k* = −11→11
*T*_min_ = 0.708, *T*_max_ = 0.819	*l* = −18→18
16794 measured reflections	

### Refinement

**Table:**

Refinement on *F*^2^	Primary atom site location: structure-invariant direct methods
Least-squares matrix: full	Secondary atom site location: difference Fourier map
*R*[*F*^2^ > 2σ(*F*^2^)] = 0.026	Hydrogen site location: inferred from neighbouring sites
*wR*(*F*^2^) = 0.069	H atoms treated by a mixture of independent and constrained refinement
*S* = 1.05	*w* = 1/[σ^2^(*F*_o_^2^) + (0.0301*P*)^2^ + 3.5121*P*] where *P* = (*F*_o_^2^ + 2*F*_c_^2^)/3
3738 reflections	(Δ/σ)_max_ = 0.002
203 parameters	Δρ_max_ = 0.54 e Å^−^^3^
0 restraints	Δρ_min_ = −0.31 e Å^−^^3^

### Special details

**Table:**

Geometry. Bond distances, angles *etc*. have been calculated using the rounded fractional coordinates. All su's are estimated from the variances of the (full) variance-covariance matrix. The cell e.s.d.'s are taken into account in the estimation of distances, angles and torsion angles
Refinement. Refinement of *F*^2^ against ALL reflections. The weighted *R*-factor *wR* and goodness of fit *S* are based on *F*^2^, conventional *R*-factors *R* are based on *F*, with *F* set to zero for negative *F*^2^. The threshold expression of *F*^2^ > σ(*F*^2^) is used only for calculating *R*-factors(gt) *etc*. and is not relevant to the choice of reflections for refinement. *R*-factors based on *F*^2^ are statistically about twice as large as those based on *F*, and *R*- factors based on ALL data will be even larger.

### Fractional atomic coordinates and isotropic or equivalent isotropic displacement parameters (Å^2^)

**Table:**

	*x*	*y*	*z*	*U*_iso_*/*U*_eq_	Occ. (<1)
I1A	0.12215 (4)	0.55411 (16)	0.41289 (12)	0.0562 (2)	0.750
I1B	0.12364 (17)	0.5371 (5)	0.4021 (4)	0.0789 (10)	0.250
Cl1	−0.04930 (3)	1.21242 (9)	0.10878 (7)	0.0669 (3)	
S1	0.16694 (2)	0.68574 (6)	0.21704 (5)	0.0391 (2)	
O1	0.18866 (8)	0.7712 (2)	0.15530 (16)	0.0565 (7)	
O2	0.18272 (7)	0.52454 (19)	0.23828 (15)	0.0482 (6)	
O3	0.13053 (10)	1.2455 (2)	0.1957 (2)	0.0776 (9)	
O4	0.05294 (9)	1.3743 (2)	0.13943 (18)	0.0690 (8)	
N1	0.10171 (8)	0.6828 (2)	0.16552 (17)	0.0441 (7)	
C1	0.18390 (9)	0.7924 (3)	0.33142 (18)	0.0361 (7)	
C2	0.16797 (10)	0.7500 (3)	0.4100 (2)	0.0418 (8)	
C3	0.18262 (12)	0.8446 (4)	0.4949 (2)	0.0556 (10)	
C4	0.21208 (13)	0.9786 (4)	0.5010 (3)	0.0624 (11)	
C5	0.22800 (12)	1.0202 (3)	0.4236 (3)	0.0578 (10)	
C6	0.21433 (10)	0.9275 (3)	0.3391 (2)	0.0453 (8)	
C7	0.06742 (10)	0.8137 (2)	0.15009 (18)	0.0371 (7)	
C8	0.08656 (10)	0.9658 (2)	0.15617 (19)	0.0402 (7)	
C9	0.05220 (10)	1.0940 (3)	0.14506 (18)	0.0394 (7)	
C10	−0.00221 (11)	1.0655 (3)	0.12406 (19)	0.0436 (8)	
C11	−0.02139 (11)	0.9137 (3)	0.1157 (2)	0.0490 (8)	
C12	0.01314 (10)	0.7883 (3)	0.1292 (2)	0.0451 (8)	
C13	0.07726 (12)	1.2528 (3)	0.1587 (2)	0.0456 (8)	
H1	0.08656	0.59352	0.14579	0.0529*	
H3	0.17239	0.81700	0.54829	0.0667*	
H3O	0.1464 (16)	1.322 (5)	0.207 (3)	0.0931*	
H4	0.22130	1.04151	0.55811	0.0749*	
H5	0.24800	1.11102	0.42821	0.0695*	
H6	0.22545	0.95503	0.28681	0.0544*	
H8	0.12289	0.98281	0.16784	0.0482*	
H11	−0.05802	0.89606	0.10069	0.0588*	
H12	−0.00012	0.68681	0.12427	0.0541*	

### Atomic displacement parameters (Å^2^)

**Table:**

	*U*^11^	*U*^22^	*U*^33^	*U*^12^	*U*^13^	*U*^23^
I1A	0.0535 (3)	0.0462 (3)	0.0796 (5)	−0.0084 (2)	0.0373 (3)	0.0109 (4)
I1B	0.106 (2)	0.0709 (18)	0.0809 (13)	−0.0395 (13)	0.0591 (12)	−0.0108 (9)
Cl1	0.0705 (5)	0.0508 (4)	0.0822 (5)	0.0268 (3)	0.0318 (4)	0.0054 (4)
S1	0.0453 (3)	0.0250 (3)	0.0528 (4)	0.0010 (2)	0.0250 (3)	−0.0009 (2)
O1	0.0748 (13)	0.0452 (10)	0.0659 (13)	−0.0060 (9)	0.0452 (11)	−0.0017 (9)
O2	0.0482 (10)	0.0272 (8)	0.0719 (13)	0.0059 (7)	0.0255 (9)	−0.0022 (8)
O3	0.0640 (15)	0.0245 (9)	0.140 (2)	−0.0043 (9)	0.0331 (15)	−0.0062 (11)
O4	0.0735 (14)	0.0240 (9)	0.0982 (17)	0.0068 (9)	0.0190 (12)	0.0050 (9)
N1	0.0451 (11)	0.0202 (8)	0.0602 (14)	0.0000 (8)	0.0119 (10)	−0.0022 (8)
C1	0.0333 (11)	0.0271 (10)	0.0489 (14)	0.0007 (8)	0.0166 (10)	0.0000 (9)
C2	0.0379 (13)	0.0389 (12)	0.0531 (15)	0.0018 (10)	0.0221 (11)	0.0042 (11)
C3	0.0577 (17)	0.0623 (18)	0.0538 (17)	0.0023 (14)	0.0290 (14)	−0.0012 (13)
C4	0.0660 (19)	0.0542 (17)	0.0633 (19)	−0.0021 (14)	0.0198 (16)	−0.0204 (14)
C5	0.0598 (18)	0.0364 (14)	0.073 (2)	−0.0110 (12)	0.0200 (16)	−0.0077 (13)
C6	0.0444 (14)	0.0344 (12)	0.0583 (16)	−0.0061 (10)	0.0205 (12)	0.0012 (11)
C7	0.0459 (13)	0.0241 (10)	0.0378 (12)	0.0028 (9)	0.0115 (10)	0.0001 (9)
C8	0.0458 (13)	0.0255 (10)	0.0476 (14)	0.0015 (9)	0.0155 (11)	0.0015 (9)
C9	0.0551 (15)	0.0246 (10)	0.0376 (13)	0.0035 (9)	0.0163 (11)	0.0014 (9)
C10	0.0545 (15)	0.0362 (12)	0.0398 (13)	0.0129 (10)	0.0172 (11)	0.0012 (10)
C11	0.0456 (14)	0.0440 (14)	0.0541 (16)	0.0017 (11)	0.0147 (12)	−0.0001 (12)
C12	0.0465 (14)	0.0310 (11)	0.0539 (15)	−0.0024 (10)	0.0143 (12)	0.0006 (10)
C13	0.0632 (17)	0.0253 (11)	0.0490 (15)	0.0040 (10)	0.0218 (13)	0.0007 (10)

### Geometric parameters (Å, °)

**Table:**

I1A—C2	2.082 (3)	C4—C5	1.370 (5)
I1B—C2	2.150 (5)	C5—C6	1.374 (4)
Cl1—C10	1.731 (3)	C7—C12	1.380 (4)
S1—O1	1.423 (2)	C7—C8	1.388 (3)
S1—O2	1.4403 (17)	C8—C9	1.399 (3)
S1—N1	1.614 (2)	C9—C10	1.388 (4)
S1—C1	1.775 (3)	C9—C13	1.494 (4)
O3—C13	1.318 (4)	C10—C11	1.384 (4)
O4—C13	1.201 (3)	C11—C12	1.379 (4)
O3—H3O	0.76 (4)	C3—H3	0.9300
N1—C7	1.409 (3)	C4—H4	0.9300
N1—H1	0.8600	C5—H5	0.9300
C1—C6	1.392 (4)	C6—H6	0.9300
C1—C2	1.387 (4)	C8—H8	0.9300
C2—C3	1.386 (4)	C11—H11	0.9300
C3—C4	1.373 (5)	C12—H12	0.9300
			
I1A···O2	3.446 (2)	C1···C8	3.215 (3)
I1A···N1	3.540 (3)	C2···C4^ii^	3.552 (5)
I1A···Cl1^i^	3.4575 (16)	C4···C2^ii^	3.552 (5)
I1A···C5^ii^	3.851 (4)	C4···O1^x^	3.293 (4)
I1B···O2	3.271 (6)	C5···I1A^ii^	3.851 (4)
I1B···N1	3.438 (6)	C5···I1B^ii^	3.835 (6)
I1B···C5^ii^	3.835 (6)	C6···C8	3.445 (4)
I1B···Cl1^i^	3.381 (5)	C6···O2^xi^	3.419 (3)
I1A···H12^iii^	3.2900	C7···Cl1^v^	3.549 (3)
I1A···H11^iii^	3.3600	C8···O1	3.193 (3)
Cl1···O4	2.939 (3)	C8···C6	3.445 (4)
Cl1···I1A^iv^	3.4575 (16)	C8···C1	3.215 (3)
Cl1···I1B^iv^	3.381 (5)	C9···C11^v^	3.499 (4)
Cl1···C7^v^	3.549 (3)	C10···C10^iii^	3.552 (4)
S1···H3O^vi^	3.15 (4)	C11···C11^iii^	3.568 (4)
S1···H8	2.7800	C11···C9^v^	3.499 (4)
O1···C8	3.193 (3)	C1···H8	2.8100
O1···C4^vii^	3.293 (4)	C6···H8	2.7700
O2···O3^vi^	2.714 (3)	C13···H1^ix^	2.9400
O2···I1B	3.271 (6)	H1···O4^vi^	2.0700
O2···I1A	3.446 (2)	H1···C13^vi^	2.9400
O2···C6^viii^	3.419 (3)	H1···H12	2.3500
O3···O2^ix^	2.714 (3)	H3···O3^x^	2.7800
O4···N1^ix^	2.903 (3)	H3O···S1^ix^	3.15 (4)
O4···Cl1	2.939 (3)	H3O···O2^ix^	1.95 (4)
O1···H8	2.5700	H4···O1^x^	2.4800
O1···H4^vii^	2.4800	H5···O1^xi^	2.7600
O1···H6	2.3600	H6···O1	2.3600
O1···H5^viii^	2.7600	H6···O2^xi^	2.6700
O2···H6^viii^	2.6700	H8···S1	2.7800
O2···H3O^vi^	1.95 (4)	H8···O1	2.5700
O3···H3^vii^	2.7800	H8···O3	2.2800
O3···H8	2.2800	H8···C1	2.8100
O4···H1^ix^	2.0700	H8···C6	2.7700
N1···I1A	3.540 (3)	H11···I1A^iii^	3.3600
N1···I1B	3.438 (6)	H12···H1	2.3500
N1···O4^vi^	2.903 (3)	H12···I1A^iii^	3.2900
			
O1—S1—O2	117.89 (12)	C8—C9—C10	118.2 (2)
O1—S1—N1	110.08 (12)	C8—C9—C13	117.2 (2)
O1—S1—C1	106.41 (12)	C10—C9—C13	124.6 (2)
O2—S1—N1	105.12 (11)	Cl1—C10—C9	123.3 (2)
O2—S1—C1	110.15 (12)	Cl1—C10—C11	116.3 (2)
N1—S1—C1	106.73 (12)	C9—C10—C11	120.4 (3)
C13—O3—H3O	118 (3)	C10—C11—C12	120.8 (3)
S1—N1—C7	125.69 (16)	C7—C12—C11	119.9 (2)
C7—N1—H1	117.00	O3—C13—O4	122.7 (3)
S1—N1—H1	117.00	O3—C13—C9	111.8 (2)
S1—C1—C6	116.10 (19)	O4—C13—C9	125.5 (3)
S1—C1—C2	124.0 (2)	C2—C3—H3	120.00
C2—C1—C6	119.9 (2)	C4—C3—H3	120.00
I1A—C2—C1	125.8 (2)	C3—C4—H4	120.00
I1A—C2—C3	115.4 (2)	C5—C4—H4	120.00
C1—C2—C3	118.9 (3)	C4—C5—H5	120.00
I1B—C2—C1	120.5 (2)	C6—C5—H5	120.00
I1B—C2—C3	120.7 (3)	C1—C6—H6	120.00
C2—C3—C4	120.7 (3)	C5—C6—H6	120.00
C3—C4—C5	120.5 (3)	C7—C8—H8	119.00
C4—C5—C6	119.8 (3)	C9—C8—H8	119.00
C1—C6—C5	120.3 (3)	C10—C11—H11	120.00
C8—C7—C12	119.5 (2)	C12—C11—H11	120.00
N1—C7—C8	122.2 (2)	C7—C12—H12	120.00
N1—C7—C12	118.31 (19)	C11—C12—H12	120.00
C7—C8—C9	121.2 (3)		
			
O1—S1—N1—C7	−65.6 (2)	C3—C4—C5—C6	0.1 (5)
O2—S1—N1—C7	166.5 (2)	C4—C5—C6—C1	0.7 (5)
C1—S1—N1—C7	49.5 (2)	N1—C7—C8—C9	−177.1 (2)
O1—S1—C1—C2	177.4 (2)	C12—C7—C8—C9	2.3 (4)
O1—S1—C1—C6	−1.6 (2)	N1—C7—C12—C11	178.7 (2)
O2—S1—C1—C2	−53.8 (3)	C8—C7—C12—C11	−0.7 (4)
O2—S1—C1—C6	127.3 (2)	C7—C8—C9—C10	−2.3 (4)
N1—S1—C1—C2	59.8 (3)	C7—C8—C9—C13	176.6 (2)
N1—S1—C1—C6	−119.1 (2)	C8—C9—C10—Cl1	179.79 (19)
S1—N1—C7—C8	15.6 (4)	C8—C9—C10—C11	0.8 (4)
S1—N1—C7—C12	−163.8 (2)	C13—C9—C10—Cl1	1.0 (4)
S1—C1—C2—I1A	0.7 (4)	C13—C9—C10—C11	−178.1 (2)
S1—C1—C2—C3	−178.5 (2)	C8—C9—C13—O3	−10.3 (3)
C6—C1—C2—I1A	179.6 (2)	C8—C9—C13—O4	170.0 (3)
C6—C1—C2—C3	0.4 (4)	C10—C9—C13—O3	168.5 (3)
S1—C1—C6—C5	178.0 (2)	C10—C9—C13—O4	−11.2 (4)
C2—C1—C6—C5	−0.9 (4)	Cl1—C10—C11—C12	−178.3 (2)
I1A—C2—C3—C4	−178.9 (3)	C9—C10—C11—C12	0.8 (4)
C1—C2—C3—C4	0.4 (5)	C10—C11—C12—C7	−0.8 (4)
C2—C3—C4—C5	−0.7 (5)		

Symmetry codes: (i) −*x*, *y*−1, −*z*+1/2; (ii) −*x*+1/2, −*y*+3/2, −*z*+1; (iii) −*x*, *y*, −*z*+1/2; (iv) −*x*, *y*+1, −*z*+1/2; (v) −*x*, −*y*+2, −*z*; (vi) *x*, *y*−1, *z*; (vii) *x*, −*y*+2, *z*−1/2; (viii) −*x*+1/2, *y*−1/2, −*z*+1/2; (ix) *x*, *y*+1, *z*; (x) *x*, −*y*+2, *z*+1/2; (xi) −*x*+1/2, *y*+1/2, −*z*+1/2.

### Hydrogen-bond geometry (Å, °)

**Table:**

*D*—H···*A*	*D*—H	H···*A*	*D*···*A*	*D*—H···*A*
N1—H1···O4^vi^	0.86	2.07	2.903 (3)	164
O3—H3O···O2^ix^	0.76 (4)	1.95 (4)	2.714 (3)	176 (5)
C4—H4···O1^x^	0.93	2.48	3.293 (4)	146
C6—H6···O1	0.93	2.36	2.792 (3)	108
C8—H8···O1	0.93	2.57	3.193 (3)	125
C8—H8···O3	0.93	2.28	2.631 (3)	102

Symmetry codes: (vi) *x*, *y*−1, *z*; (ix) *x*, *y*+1, *z*; (x) *x*, −*y*+2, *z*+1/2.
